# Isolating the effect of cycling on local business environments in London

**DOI:** 10.1371/journal.pone.0209090

**Published:** 2018-12-20

**Authors:** Konstantin Klemmer, Tobias Brandt, Stephen Jarvis

**Affiliations:** 1 Warwick Institute for the Science of Cities, University of Warwick, Coventry, United Kingdom; 2 Department of Computer Science, University of Warwick, Coventry, United Kingdom; 3 The Alan Turing Institute, The British Library, London, United Kingdom; 4 Rotterdam School of Management, Erasmus University, PA Rotterdam, The Netherlands; University of Sydney, AUSTRALIA

## Abstract

We investigate whether increasing cycling activity affects the emergence of new local businesses. Historical amenity data from OpenStreetMap is used to quantify change in shop and sustenance amenity counts. We apply an instrumental variable framework to investigate a causal relationship and to account for endogeneity in the model. Measures of cycling infrastructure serve as instruments. The impact is evaluated on the level of 4835 Lower Super Output Areas in Greater London. Our results indicate that an increase in cycling trips significantly contributes to the emergence of new local shops and businesses. Limitations regarding data quality, zero-inflation and residual spatial autocorrelation are discussed. While our findings correspond to previous investigations stating positive economic effects of cycling, we advance research in the field by providing a new dataset of unprecedented high granularity and size. Furthermore, this is the first study in cycling research looking at business amenities as a measure of economic activity. The insights from our analysis can enhance understandings of how cycling affects the development of local urban economies and may thus be used to assess and evaluate transport policies and investments. Beyond this, our study highlights the value of open data in city research.

## 1. Introduction

The transportation sector is one of the major factors that powers a thriving economy. Ever since the first human civilizations started trading, the global economic system has crucially depended upon transport infrastructure and its adaptation to new requirements and needs [1]. Today, especially urban areas rely on sophisticated, multimodal transportation networks to meet travellers’ capacity and connectivity requirements. The rise of new technologies has helped to improve existing transportation infrastructure and enabled new means, such as, for instance, electric vehicles or shared mobility concepts. These developments have also given rise to the idea of ‘smart cities’, describing the interconnection among physical and non-physical environments and their role in shaping urban performance [2]. With the increasing digitisation of cities comes large volumes of continually produced data [3]. The urban data revolution holds great potential for decision makers, allowing them to quantify, model and improve the urban landscape. This process however is not without criticism. Previous research, for instance, has described the ‘smart cities’ paradigm as contradictory to the informal character of cities, and driven by capitalist, profit-oriented ideas which lead to a reproduction and reinforcement of urban inequalities [4]. Rob Kitchin proposed concrete changes, calling for a re-orientation of the urban conception, a re-configuration of epistemology and the adaption of ethical principles in policy making [5]. But even though most of the discussion surrounding the ‘smart cities’ agenda addresses technological opportunities and challenges, not all trends in urban transportation are purely technology-driven: In the light of congestion and rising popularity of active lifestyles, cycling has become more and more prevalent [6]. Although politicians and transport authorities increasingly promote cycling adoption to ease urban transportation overload [7], little is yet known about the consequences on the economy and urban development. This emphasises the necessity for scientific research to examine this area, revealing underlying dynamics and causalities.

We see this research as at the intersection of two developments: Connecting newly available, detailed open data to investigate how cycling adoption affects the urban business landscape. Whilst previous research has rigorously analysed the economic impacts of road and rail transport, cycling’s effects have thus far attracted little research interest. The lack of available studies in combination with growing political support makes this research especially relevant. With respect to this development, more sophisticated insights on the connection between urban cycling and the economy may help with planning and appraisal of cycling infrastructure projects. The primary aim of this paper is hence to address the question of *whether*, *and to what extent*, *increased cycling activity has led to the emergence of new local businesses*. We address this question in the urban setting of Greater London.

One of the main reasons for the lack of cycling-related research is the difficulty of assessing its marginal effect within the broader multimodal urban transportation system, where cycling plays only a minor role. We tackle this challenge by applying a new, highly granular dataset within a sophisticated statistical framework to *isolate* the economic impact of cycling infrastructure expansion. The city of London offers a promising environment in which to conduct this project, as it is characterised by a thriving open-data landscape and a strong ambition to become more bike-friendly [8]. Hence, a secondary aim of this project is to evaluate the applicability of open-data for analysing human movement patterns and investigating how transportation paradigms shape the urban socio-economic landscape. In this regard, this paper serves as a case-study and may encourage more data-driven research and policy to the benefit of cities around the world.

The novelty of this paper’s contribution is two-fold: (1) We compile a completely new dataset assembled entirely out of open data provided by public authorities and the community mapping service OpenStreetMap (OSM). (2) Furthermore, we are the first researchers to relate cycling activity to new shop openings—thus capturing both economic and urban development. The first part of the paper will review previous literature on the matter and contextualise new research opportunities. Subsequently, we will introduce the dataset specifically compiled for this project, combining and standardising sources from various open-data providers on a common geographical level. Next, we construct a methodological framework for statistical analysis, taking into account the negative-binomial nature of the gathered data and imposing treatments for data shortcomings and endogeneity, thus aiming to establish a causal relationship between growth in cycling and local businesses, but also taking into account potential limitations. We then present our results, commenting on the validity of the obtained findings. Finally, we summarise the results of our research and describe its potential policy implications. We conclude the paper with a research outlook addressing potential directions for future work.

### 2. Review of the literature

Our literature review is divided into three parts. First, we synthesise previous research on the economic effects of the transport sector—both broadly and with a focus on cycling. Second, we explore the use of urban amenity data for studies in the fields of economics and transportation research. Third, we address the application of open data for project appraisal and policy evaluation. To the best of our knowledge, these three fields have not yet been reflected jointly, presenting the opportunity to extend the current state of research with our contribution.

### 2.1. Economic effects of transport interventions

The main motivation for examining the economic impact of transportation is the integration of the gathered insights into infrastructure project appraisal frameworks. Thus, academia can serve the public and private sector with valuable tools for planning and decision making. It is hence critical to understand the exact interplay between a transportation system and its surrounding environments. Lakshmanan [9] presents an overview of previously used methodologies in assessing economic effects of transport infrastructure improvements and highlights that economic effects play out in various forms and interactions, which may be integrated in economic equilibrium models. Generally, research in the field can be categorised into three topic areas: accessibility and land-use, productivity and labour markets, as well as spatial economics and local effects.

Accessibility and land-use research seeks to investigate the effect of transport projects on connectivity and the use and valuation on building land. Despite the general difficulty of deriving reliable accessibility measures [10], current literature has drawn clear links between the characteristics of the transportation sector and land-use. For example, links between transport investment and rising land- and property values have been widely analysed and acknowledged [11–13]. The effects however appear to depend on the characteristics of an area, or upon issues such as the urban-rural divide. Another widely reviewed field is the connection of transportation and labour, particularly productivity and employment. Private and public transport is crucial in moving the workforce from dwellings to their respective workplaces. Expansions in transport systems can not only improve labour market accessibility, but also intrinsically stimulate employment. Recent literature has shown that employers specifically consider transport infrastructure when choosing the location of manufacturing sites [14]. Notably, Graham [15] isolates positive productivity externalities arising from agglomeration and transport investment in urban areas. His study again emphasised that the outcome of transport interventions is highly location-sensitive and must be tailored to fit the treatment region. Indeed, spatial economics and local characteristics seem to play a major role in driving economic effects and their extent. Moreno and López-Bazo [16] argue that local infrastructure investments (e.g. electric grid or broadband infrastructure) prove more efficient than transport infrastructure investments in terms of return on capital. On the other hand, Gibbons and Machin [17] show that local rail innovations are highly valued by surrounding households. This can not only be observed in an increase in housing prices, but also the valuation of other local amenities. Overall, previous research suggests a positive economic effect of transport infrastructure improvements. Cities seem to experience multiplicative effects, attributed to densification and agglomeration.

Cycling has received far less attention from academics compared to other modes of transport. Nevertheless, rising popularity and newly available data has enabled more thorough approaches to assessing the beneficial economic effects of cycling, as for instance outlined by Flusche [18]. The author presents four main aspects:

1. The economy around the bike itself (e.g. bicycle shops and repair workshops)
2. Revenue gains for businesses profiting from increasing cycling accessibility
3. Revenue gains for businesses from conventional bike use and repeated trips
4. Economic benefits from cycling tourism

Additionally, the author argues that cycling also saves money by lowering travel costs, decreasing corporate health insurance, and cheapening bicycle parking. However, this still only addresses *direct* effects of cycling. Spillover effects also appear to be highly significant: Cycling has been shown to have positive effects on many factors of physical health, which outweigh the adverse effects of cycling in polluted urban areas [19,20]. Further positive externalities are cyclists’ contributions to resolving congestion [21] and to the reduction of air pollution [22]. It can thus be assumed that—even if more sophisticated studies are just beginning to emerge—cycling generates significant and positive social benefit. This is of course only the case if the promotion of cycling, e.g. via the expansion of infrastructure, also leads to an increase in bike use. Previous research has shown that proximity to cycling infrastructure is indeed a determinant in bike travel adoption [23]. Furthermore, travellers perceive cycling as less stressful and more enjoyable than other modes [24]. With the economic benefits of cycling and the links between cycling infrastructure and adoption being established, we now briefly review literature on the infrastructure measures addressed in this project: bike-sharing schemes and bike parking.

As research on bike-sharing mainly addresses the effects on health, there is very little research on direct economic effects. In a recent study, Pelechrinis et al. [25] estimate a positive impact of shared bicycle systems on housing prices. Bike-sharing has also been associated with higher retail shopping activity [26,27]. Nonetheless, most evidence concerns the effect of bike-sharing on other modes, specifically a decrease in road traffic and complementary use with public transport [28,29]. More generally speaking, Médard de Chardon et al. [30] argue, that the success of a bike-sharing scheme critically depends on network effects. Jäppinen et al. [31] predict that the introduction of a bike-sharing scheme in Helsinki would lead to a 10% decrease in public transport travel times. Bullock et al. assess the wider economic effects of bike-sharing in Dublin and conduct a detailed cost-benefit analysis, highlighting the overall positive effects of the scheme [32]. It is worth noting here, however, that bike-sharing is not uncontroversial. Critics have raised concerns about the equitability of cycling in general and bike-sharing in particular. Indeed, Stehlin describes urban cycling as both a “vector and symbol of gentrification”[33]. Flanagan et al. show that underprivileged communities are less likely to attract funding for cycling infrastructure [34]. Indeed, cycling in the UK remains an activity for mostly white and male individuals [35]. However, increasing efforts to make cycling more equitable have been evident in recent years [36]. London is at the forefront of these movements with several active organisations promoting cycling among underrepresented communities, for instance providing free bikes or cycling classes [37].

Turning to bicycle parking infrastructure, Buehler [38] has shown that providing free bike parking increases commuting activity by bike. McNeil [39] explores how cycling accessibility is improved by expanding parking infrastructure in a case study of Portland. He argues that thoroughly planned infrastructure projects could increase cyclists’ connectivity to stores, restaurants and other potential destinations, hence stimulating both bike use and local businesses’ revenue. McNeil also makes the point that urban amenities are of crucial importance for travelling. We follow a similar approach and investigate further recent literature in this field within the next section.

### 2.2. The role of urban amenities in understanding cities

Amenities reflect the demography, economy and culture of a city and as a result, are the essential determinants of how residents perceive their urban environment [40]. Even though we will focus on physical amenities (e.g. stores and restaurants) in this paper, the term ‘amenity’ also refers to more intangible concepts, such as air quality [41]. As such, amenities are interesting for many multidisciplinary research questions. In economics, amenity data has for instance been utilised in analysing urban migration patterns [42] and assessing property values [43,44]. For the most part, this study was concerned with urban retail businesses—which can be described as consumption amenities. In a recent paper, Kuang [45] showed that local consumption amenities contribute to the attractiveness of a neighbourhood. We will address more literature on consumption amenities in section 4.4, where we discuss potential exogenous drivers of new business openings. Transportation research has also shown increasing interest in amenity data. A recent paper by Hu et al. [46] suggests that due to its high granularity and geographical reference, amenity data improves accuracy in urban modelling. Indeed, physical amenities have been proven to be valuable in explaining spatio-temporal patterns in urban carsharing usage [47] or the perception of transit waiting times [48]. The availability and quality of urban amenity data has vastly increased over the last few years, which can be attributed to the previously mentioned trend of public and private data democratisation known as open data. However, present literature is lacking representation of the relationship between urban amenities and the cycling environment. While the few papers raising this question are mostly concerned with the interconnection of physical structures and cycling adoption, we did not come across an approach that uses amenity data as a measure of both, economic activity and cycling attractiveness.

### 2.3. Open data for project appraisal and policy evaluation

Key characteristics of *smart city* initiatives include the quantity, quality and accessibility of their data ecosystems. While such projects often address many different domains (e.g. economy, energy and education), their main purpose is leveraging public and private actors, eventually sparking urban innovation [49]. Schaffers et al. [50] regard open data as one of the main drivers of innovation within urban collaboration frameworks. Nonetheless, the execution of open data strategies is particularly important, as Janssen et al. [51] argued in a study laying out the potential and challenges. The authors make the point that the release of open data often goes along with unrealistic expectations, sometimes caused by disregard of the user perspective. The availability and ease-of-use was discussed in-depth by Arribas-Bel [52], naming open data among mobility data and online service provider data as a key source for a deeper understanding of cities. As a consequence, research, public policy and corporate decision making will be increasingly data driven. Einav and Levin [53] lay down the potential use of data for public administration issues (e.g. taxation and healthcare management). Economic research—regularly consulted by policy makers—will profit in two ways: On the one hand by obtaining larger, more detailed datasets for quantitative analysis, on the other hand by enabling new methodologies, such as leveraging the analytical frameworks developed in emerging fields like machine learning and data science.

## 3. Data

### 3.1. Data sources

As mentioned earlier, the complexity and noise of urban environments complicates the observation of peripheral factors, such as cycling. Yet, our approach is fundamentally driven by novel, emerging data sources which allow us to address this complex problem. In specifying the research question, we first identify the required domains from which we seek to extract data. We then aim to analyse the effect of (I) *cycling usage* on the (II) *emergence (i*.*e*. *openings) of local businesses*, taking into account measures of (III) *cycling infrastructure* and controlling for (IV) *socio-economic and demographic factors*.

- While it is difficult to gather high quality data on the use of private bikes, many cities around the world have installed increasingly popular bike sharing systems. The London scheme is run by Transport for London (TfL), who publish detailed trip data as part of their open data strategy. This allows us to measure the attractivity of cycling over time and make comparisons between intervals.
- Local business data comes as consumption amenity locations from OSM. Services like *Geofabrik* offer OSM data backups at historical points-in-time for the Greater London area. We can hence compute the difference in tagged objects to assess changes in amenity prevalence over time. The local business data can be divided into several subcategories, including for example clothing stores or fast-food restaurants.
- To validate the arguments regarding potential effects of cycling usage, we also include measures of the broader cycling ecosystem. This enables us to treat endogeneity during the statistical modelling process (see Section 4) and eventually draw causal inference. We look at two specific measures of cycling infrastructure: bike-sharing stations and bike-parking facilities. Both are physical amenities and can likewise be extracted from *Geofabrik’s* OSM archive in a timely form. Beyond infrastructure, we assess spatio-temporal cycling accident data as provided by TfL and bicycle shop amenity data, again available via OSM. These two additional variables help us to draw a more pervasive picture of the urban cycling landscape.
- Socio-economic data for London is available from the London Datastore as part of the cities open data strategy. More precisely, we collect over 300 different factors including information on population density, employment status and ethnicity. The London Datastore also provides the geographical reference upon which we join all collected data.

### 3.2. Data processing and standardisation

**Fig 1:**
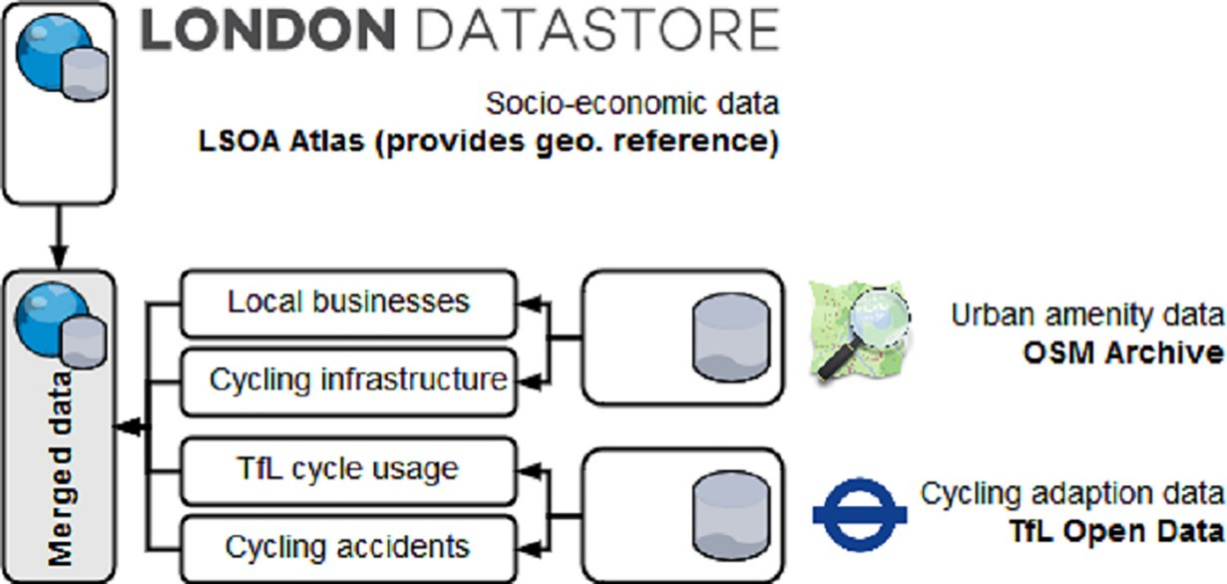
Data sources and dimensions.

Since all our data comes from different sources and in different form, we need to process and join it under a common reference framework before proceeding with any analysis (see Fig 1). First, we need to identify a common frame of reference enabling us to combine data from different sources. Looking at the city of London, we opt for Lower Super Output Areas (LSOA) as a common geographical level. These areas are polygons initially designed according to their respective population share in order to improve statistical reporting for small areas [54]. We chose the LSOA level as it comes with exhaustive census, socio-economic and demographic data. The geographical polygons allow us to join further data by their spatial dimension. The Greater London area consists out of 4835 LSOA’s.

OpenStreetMap is the largest open source mapping project on the Internet. It is a valuable tool for constructing urban networks and quantifying city structures, such as cycling infrastructure [55]. Accordingly, the use of OSM data for public policy and urban planning has been highlighted in a recent study [56]. However, following a volunteered geographic information (VGI) approach, OSM data is not always perfectly reliable [57–59]. Recent studies have also addressed the issue of fairness and representation in OSM. Calling for data equitability and a critical geography perspective, Glasze and Perkins [60] suggest that the community map might reproduce social realities and inequalities. However, Tenney [61] finds that socio-economic factors only marginally affect OSM data density and community participation in urban areas, whereas inequalities are mostly observed in rural areas. Essentially, there are three reasons why we select OSM as data source for this research: (1) Even though not perfect, data quality in OSM is still good [62]. In fact, OSM outperforms proprietary mapping services like Google Maps or Bing Maps and errors have been shown to decrease over time and with growing communities [63,64]. Over et al. [65] comment that OSM has probably the most up-to-date map data and that “[i]n urban areas, changes in the road network appear in the OSM data set long before appearing in other map providers’ data”. This holds especially true for London, where the OSM project was started in 2004 and a large community of volunteers constantly works on mapping changes in the city. An active and geographically spread out community has been shown to increase data quality [66,67]. Senaratne et al. [67] provide a comprehensive overview on OSM data quality assessment studies. (2) Working with OSM data allows us to make an assessment regarding its quality a further objective of this research project—see e.g. our comments addressing the potential of open data and our in-depth discussion of existing research above. (3) OSM is, to our knowledge, the largest geodata provider offering historical extracts. Historical mapping data can be accessed via the OSM archive at *Geofabrik*. We address our aim of observing urban amenity changes over time and testing whether the vicinity of an amenity has been affected by a change in cycling activity. Accordingly, we include extracts from the start of each of the years 2014, 2015, 2016 and 2017, to determine when within the timeframe certain amenities emerged. Importantly, we assume that the date an amenity was tagged on OSM approximates to the date when the amenity first appeared. Since we are missing precise data on when a shop or bicycle parking facility was opened, we necessarily rely upon volunteered OSM tagging dates to represent the actual opening date. Two arguments justify this assumption: First, as mentioned above, London has a thriving community of OSM volunteers being the first city to be mapped by the service, which has been shown to increase data quality. Second, we are looking at yearly data which allows for a large time buffer between tagging and actual opening (up to one full year). Overall, we believe that the evidently good OSM environment in London, the active community and the yearly aggregation provide us with a sufficiently robust data source for our study.

We filter the OSM data using a key system (see OpenStreetMap [68]) to extract required amenities. For example, shops can be accessed via *key*:*shop* and are further classified into subcategories such as *optician*, *dry_cleaning* or *supermarket* (e.g. using the tag *shop = ‘supermarket’*). We treat bicycle shops (*shop = ‘bicycle’*) separately, as they will serve as instruments for endogeneity treatment (see section 4). Other physical amenities can be accessed via the *key*:*amenity*. These are TfL cycle hire stations (*amenity = ‘bicycle_rental’* and *network = ‘tfl_cycle_hire’*), bicycle parking facilities (*amenity = ‘bicycle_parking’*) and lastly sustenance amenities (*amenity = ‘restaurant’*, *amenity = ‘bar’*, *amenity = ‘fast_food’*, *amenity = ‘pub’* and *amenity = ‘cafe’*) which we also consider as local businesses. The tagging systems enables us to investigate the effect of cycling on specific business subgroups or on an aggregate level. All shops and amenities come as geo-point data which we can easily associate with an according LSOA. We count amenity occurrences per category per LSOA. The developments of amenity counts are displayed in Table 1. The amenity data already highlights changes in amenity counts that we can examine concerning a potential mutual interaction. The number of shops, for instance, doubles over the observed period. This is due to the general delay in tagging throughout the expansion of OSM. As such, any delay bias is equally implicit for each area and hence does not impact our modelling approach (refer back to our discussion of data inequalities and community participation in OSM above).

**Table 1:** OSM amenity data: overview.

	Year			
Amenity count	2013	2014	2015	2016
TfL cycle hire station	430	445	466	482
Bicycle parking facility	3,448	3,786	4,257	4,909
Bicycle store	139	154	174	197
All shops (excl. bicyc. stores)	6,862	8,275	10,318	12,077

Further extracted amenity subcategories: *eating* & *drinking amenities*, *financial amenities*, *healthcare amenities*, *tourism amenities*, *food & drink shops*, *general shops*, *clothing* & *fashion shops*, *beauty shops*, *construction* & *furniture shops*, *electronics shops*, *sport* & *activity shops*, *book* & *gift shops*

To validate bicycle adoption, we access bike-sharing and bike accident data via TfL’s Open Data portal. Data on shared bicycle usage can be found for our observational period from 2013 to 2016. The data contains every recorded bike rental including start and end station of each trip. We can now aggregate usage per station per year and join this on LSOA level. TfL also provides the London records of traffic accidents as collected by the Department for Transport (DfT). This data comes with timestamp of occurrence and geographical location for each accident. We filter the data for incidents involving bicycles from 2013 to 2016, count accidents per year and aggregate bicycle accident counts on LSOA level. This concludes the data gathering and preparation process. Next, we outline the methodological framework.

## 4. Methodology

### 4.1. Data exploration and cleaning

**Fig 2:**
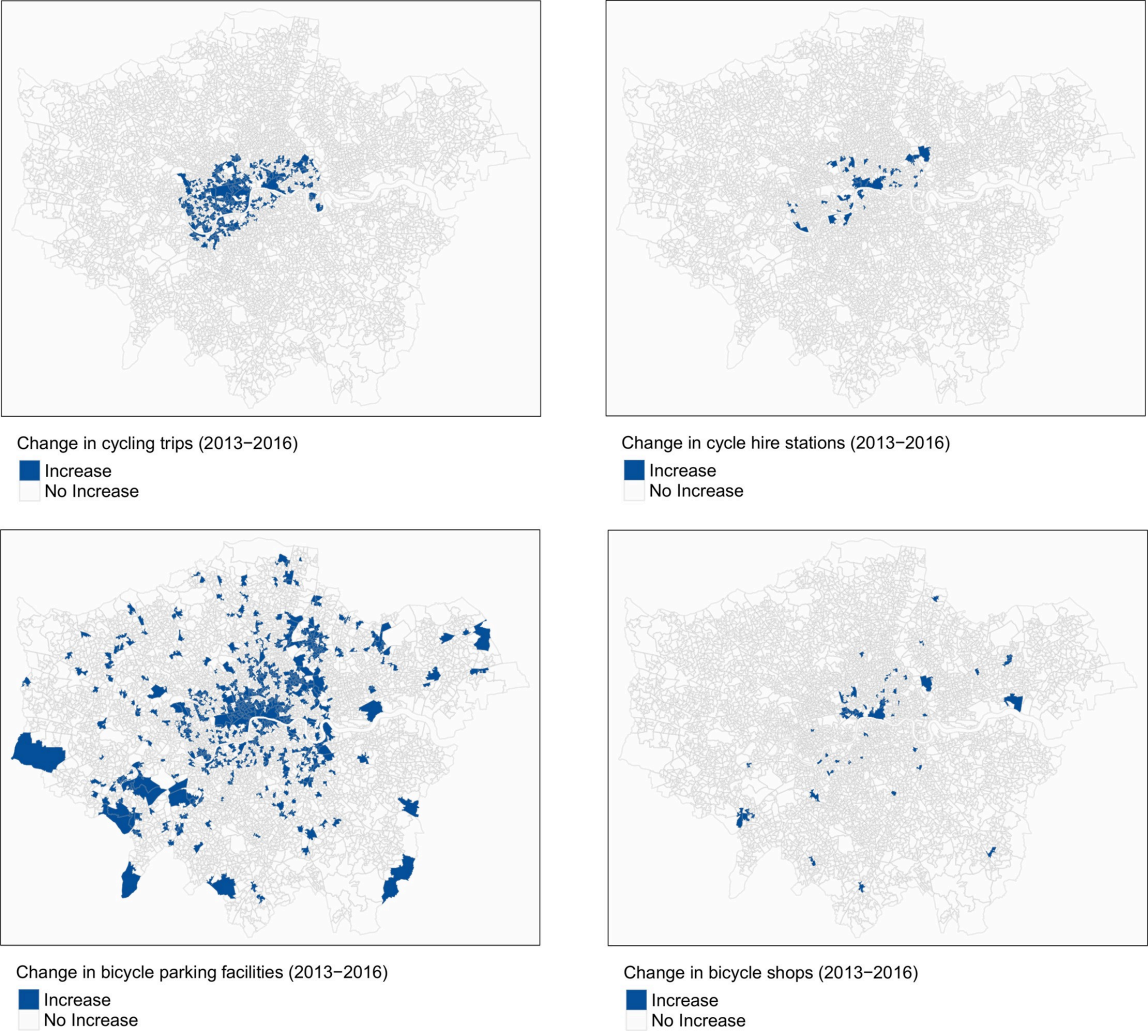
Determination of cycling activity and infrastructure changes (2013–2016) excluding intensities.

At the core of our analysis lies the comparison of areas that experienced an increase in cycling activity with areas that remain unchanged. We find that 262 LSO areas out of a total of 4,835 exhibited an increase in cycle hire trip starts between the years 2013 to 2016; 260 areas had more cycle hire trip ends. Overall, cycle hire trip start and end counts are extremely similar—which is expected as each trip end station is likewise the start station of the next trip with the same bike. We hence limit our analysis to the investigation of trip end counts. Furthermore, we also look at changes in the cycling ecosystem as illustrated in Fig 2.

Apart from bicycle parking, we observe a strong concentration of cycling activity and infrastructure in central London, where the TfL cycle hire scheme operates. Bike shops also seem to emerge mostly in central London. From this observable centrality, questions regarding spatial dependencies in our data arise. Spatial autocorrelation, i.e. the correlation of geographically neighbouring datapoints, could be a potential threat to our model quality, as it violates the assumption of independent model error terms. We apply global and local Moran’s I [69] testing procedures and find significant spatial autocorrelation in the dependent variable (difference in shops and consumption amenities). We can also observe that the autocorrelation corresponds to the centrality of our data. Spatial autocorrelation of the dependent variable in a model is not a problem per se, nevertheless it motivates us to investigate further and to test our final models for residual spatial autocorrelation (RSA). We comment on our findings and the resulting limitations more thoroughly in the discussion section.

**Fig 3:**
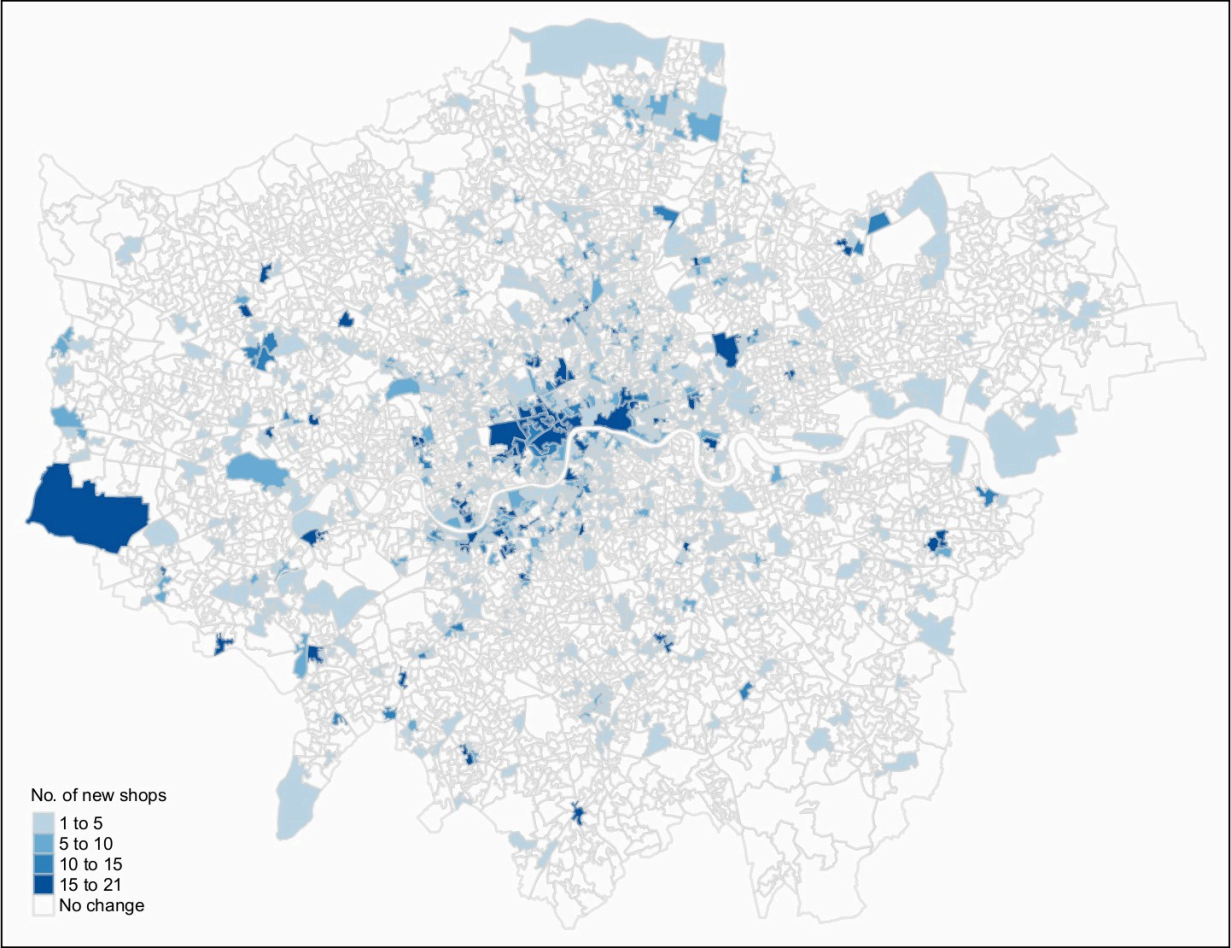
Overview of new shop counts (2013–2016) after outlier treatment.

The count data exposes a strong inflation of zero counts, which we address during our analysis. We now examine whether growth areas (areas with increased cycling activity) experience a significantly larger number of new local business openings. We have collected several different categories of local business amenities and show the growth in amenities tagged as shops (shop = *) in Fig 3. Interestingly, none of the observed areas exhibits a decrease in the number of shop counts. The number of unchanged regions is 3859 out of 4835. This can be explained by new shops often replacing old ones, limited dynamics in residential neighbourhoods and the previously mentioned characteristics of OSM.

We also find substantial outliers in shop count and bicycle parking facility differences which might harm our subsequent modelling efforts. We thus decide to treat outlier effects in both categories by fixing high counts at the 99% quantile. We then compute indicator dummies, which describe whether an area has experienced an increase in TfL cycle hire trips for an initial comparison:

$$f(x_{\left\{\Delta Cyc.trip\right\}})=\left\{ \begin{array}{c} 1,if\;x>0\\ 0,if\;x\leq 0 \end{array}\right.$$ Eq 1

The indicator dummies allow us to split our data into growth and non-growth samples. However, we cannot simply test for a difference in means between the two samples, as most standard procedures assume normally distributed data. We hence test the difference in shop counts between treated and un-treated samples for the null hypothesis *H*_0_ that the samples are normally distributed, using the Shapiro-Wilk test [70]. The results of the test are displayed in Table 2 and clearly suggest that neither sample follows a normal distribution.

**Table 2:** Shapiro-Wilk normality test for differences in shop counts by LSOA.

	Sample	W (test statistic)	p-value
**Cycling activity**	Increase	0.646	< 0.001 ***
	No Increase	0.291	< 0.001 ***

Significance codes: 0 *** 0.001 ** 0.01 * 0.05.

Note: Increased cycling activity is measured using TfL cycle hire trip end counts

As a result, we turn to a distribution that is common for count data—especially if it comes with a heavy zero inflation: that is the negative binomial (NB) distribution. The NB distribution is a discrete probability distribution with probability mass function

Pr,p(x)=(x+r−1r)pr(1−p)x Eq 2

and distribution function

D(x)=I(p;r,x+1) Eq 3

Where *I*(*z*;*a*,*b*) represents a regularised beta function. Given a sample ${\left\{x_{i}\right\}}_{i=1}^{n}$, the NB distribution describes the number of successes, occurring with probability $p=\frac{r}{\sum_{i}\;x_{i}/nr}$ in sequential Bernoulli trials before a predefined number of failures *r* is reached. Mean and variance for NB distributions are given as $\mu =\frac{rp}{1-p}$ and $Var(x)=\frac{rp}{{\left(1-p\right)}^{2}}$ respectively.

### 4.2. Sample comparison and temporal precedence

**Fig 4:**
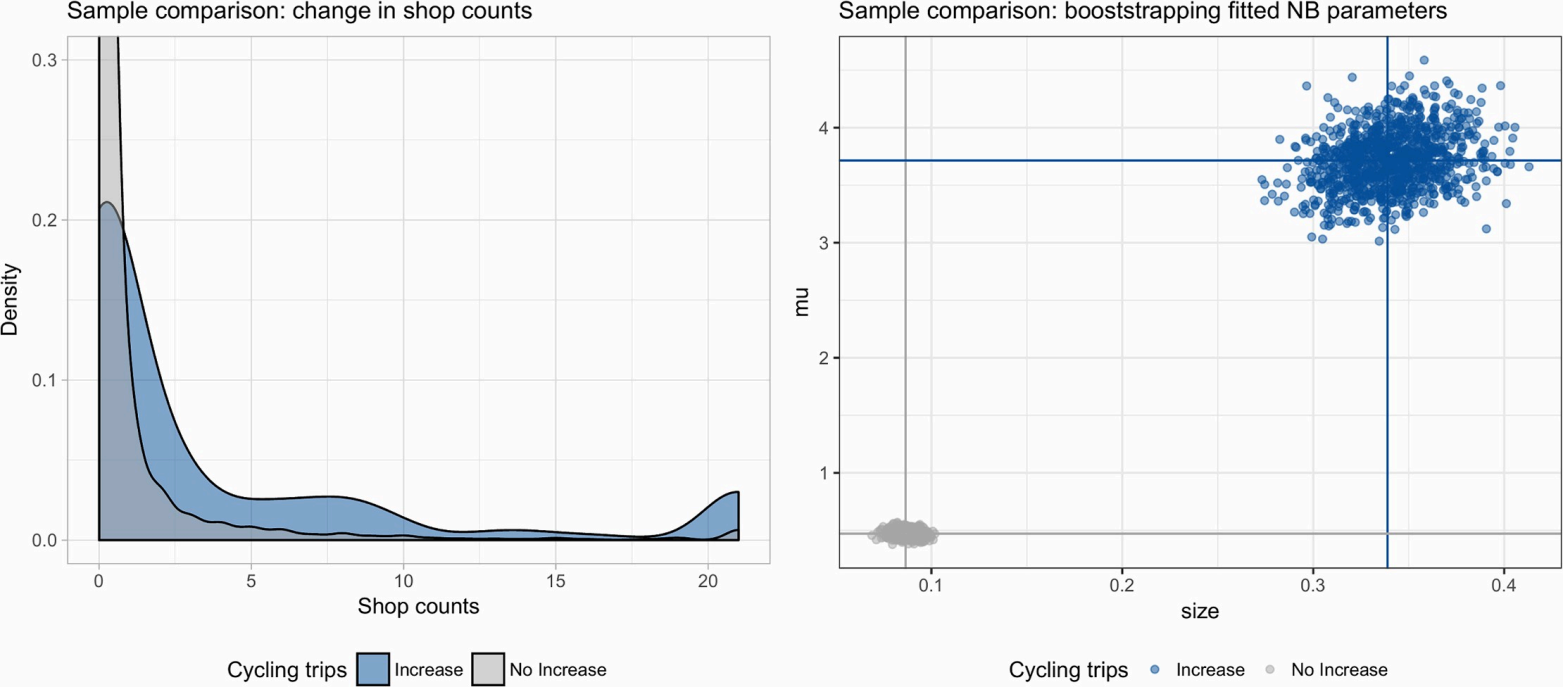
Density and bootstrapped sample comparison by cycling trips treatment. Note: bootstrapped samples are compared by mean μ and number of successes ‘size’.

NB distributed data unfortunately rules out many of the standard tests, e.g. the Welch t-test for equal sample means. However, a graphical comparison of the shop counts between treated and control areas indicates that the count density functions are rather different from each other (see Fig 4A). Note that the high density at the right tail (maximum value) comes from fixing outliers at the 0.99% quantile, as discussed above. We observe that shop count differences > 0 are considerably more frequent across the indicator group—keep in mind that LSOAs are established to represent equal population size. The indicator sample is heavily biased towards the less residential Central London which likely accounts for a considerable portion of the density differences between both samples. Nonetheless, this is the first clear indication of a positive association between an increase in cycling trips and the difference in shop counts across the observed areas. To obtain further validation, we now apply a bootstrap testing framework.

We again split our data into treatment and control samples. We apply ordinary random sampling with replacement from each sample population, where the size of the bootstrapped sample *N*_*BS*_ is equal to the size of the sample population *N*_*S*_ with *k* = 1000 repetitions. For each of the bootstrapped samples, we fit a negative binomial distribution according to its mean (*μ*) and number of successes (*size*) parameters. The results of the bootstrapping test are displayed in Fig 4B. Across all 1000 repetitions, the bootstrapped samples of treated and control data are characterised by unambiguously different NB distribution parameters. Thus, we conclude that the samples do not stem from the same distribution.

**Fig 5:**
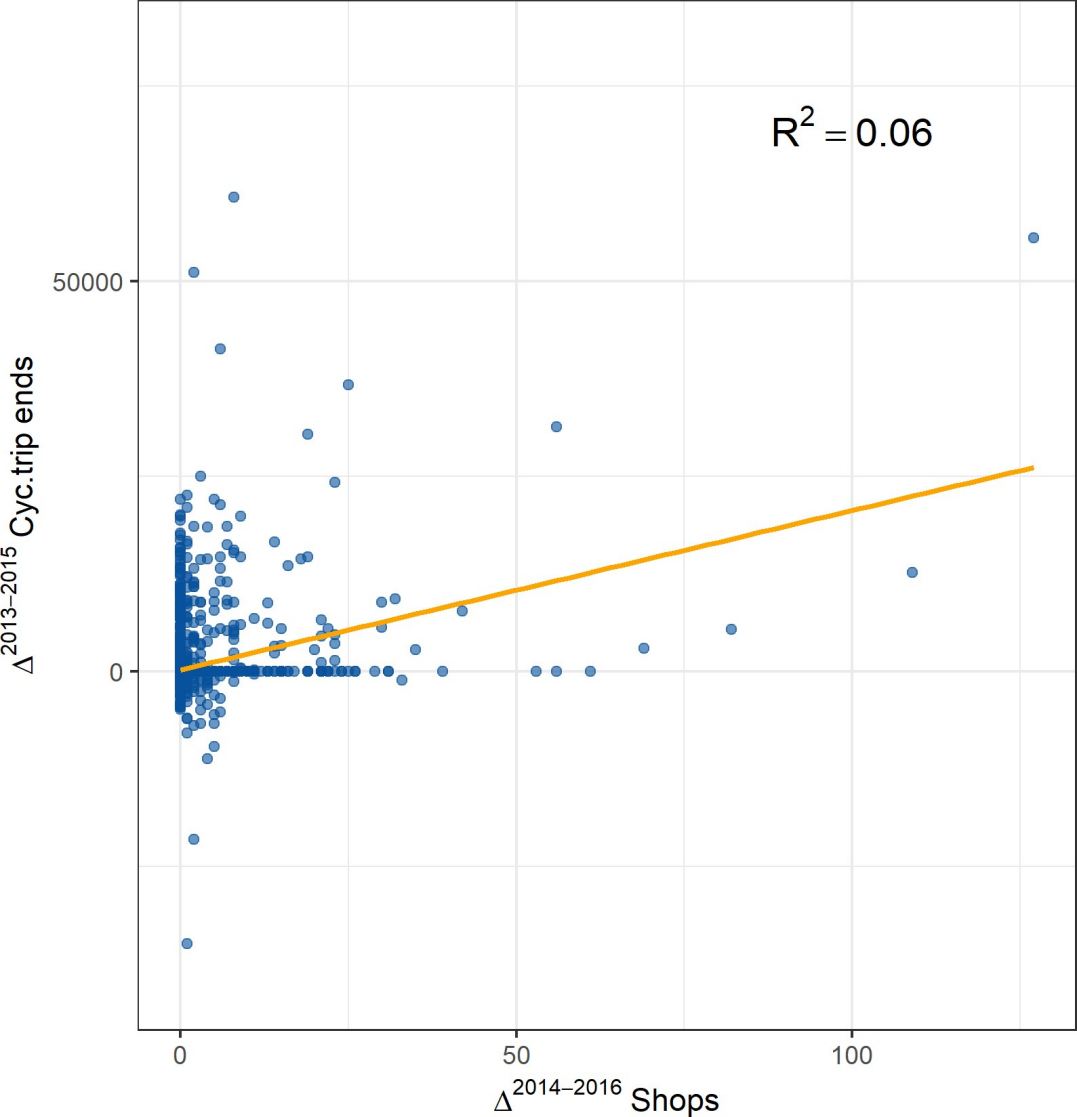
Scatterplot of a univariate linear regression with Δ^{2014−2016}^*Shops* as dependent variable and Δ^{2013−2015}^*Cyc*.*trip ends* as independent variable.

Lastly, temporal dependences might also provide a further hint at an underlying causal process and have been proven to be useful in previous bikesharing research [71]. We run several tests with lagged regression models (note that the regression procedure for negative binomial data is outlined in the following sections), where we predict change in shop counts Δ_*t*,*t*−1_*Shops* with temporally lagged changes in bicycle trip end points Δ_*t*−1,*t*−2_*Cyc*.*trip end* (see Fig 5). This helps us to examine whether a change in cycling trips precedes a change in shop counts. Across models with different lags, we find a consistent, significant and positive effect of changes in cycling trips on future changes in shop counts. This effect is confirmed for sustenance amenities.

### 4.3. Treating reverse causality

While the established association between the difference in shop counts and cycling activity might serve as incidence of causality, it is not conclusive evidence of a causal relationship between both factors. Recalling our initial research question, we want to investigate the functional relationship between the development in business amenity counts *y* and development in cycling trip counts *x*. This builds on the hypothesis that increased cycling activity incentivises local shopping by improving accessibility to the local retail ecosystem, thus motivating new business openings. In a linear model, this can be denoted as

$$y_{i}=\beta_{0}+\beta_{1}x_{i}+\epsilon_{i}$$ Eq 4

where *ϵ* describes the error term within the model, capturing all variation in the outcome variable *y* that cannot be explained with the exogenous variable *x*. However, the key problem here is the reverse causality between *x* and *y*. In other words, that an increase in cycling causes growth in newly emerging local businesses and reverse—an intuitive argument as more cyclists imply more potential customers, while more shops attract more cycling trips. This denotes *x* as an endogenous variable, i.e. implies that *x* is correlated with the error term *ϵ* which is a crucial violation of the linear model assumptions as it renders the OLS estimator inconsistent. We will provide evidence for the existence of endogeneity in the results section.

The challenge arising from this issue is to isolate the unilateral causal effect of the predictor *x* on the outcome *y*. We account for this endogeneity problem by using an instrumental variable (IV) approach (see e.g. [72]). Within our framework, we introduce an IV *z* that is correlated with the endogenous predictor *x* but is uncorrelated with the model error term *ϵ*. This is also referred to as the exclusion restriction. Unfortunately, there is no way to test for correlation between instrument and true error term—as it is unknown. Overcoming the endogeneity problem hence necessitates identifying instrumental variables *z* that are supported by strong theoretical arguments. In our particular case, we need to find some approximation measure that shows a strong correlation with new increasing cycling activity. Looking at the broader urban cycling ecosystem and our available data, we identify four promising instruments, i.e. we suspect correlation with the endogenous variable and independence from the model error term: (I) *TfL cycle hire stations*, (II) *bicycle parking facilities*, (III) *bicycle accident data* and (IV) *bicycle shops*.

*Instruments I and II*: Cycling infrastructure data comes in the form of a four-year difference in amenity counts at LSOA level. The argument for correlation with the endogenous variable is relatively straightforward. We assume that an increase in cycling infrastructure goes along with a growing attractivity of cycling, driving up cycling activity. This relationship has been proven in many scientific studies (e.g. [73]). The reasoning is of course especially strong for new TfL cycle hire interventions, but also relates to bicycle parking infrastructure. A problem here however, is the question of whether cycling infrastructure and local business emergence share a direct causal link. We argue, that the true effect is indirect and manifests itself through cycling activity. Intuitively, infrastructure can only affect local business environments if it is actually used—as shown by activity. Moreover, the Mayor’s vision for cycling in London [8] outlines an infrastructure expansion strategy: Included areas are (1) along the tube and TfL rail network, (2) in residential areas to promote commuting by bike and (3) in areas with pre-existing bicycle infrastructure, mostly along the cycle superhighways and quiteways. This explicitly tells us that TfL does not look at ongoing or anticipated local business growth when planning new cycling infrastructure. In fact, TfL’s primary interest is not short-term profit maximisation, but rather aligns with the Mayors long-term vision for London’s urban development. Beyond that, the provision of cycle hire stations is often driven by the local political agenda and partially depends on a Borough’s willingness to pay [74]. Lastly, cycle hire stations are currently required to be located within 300-meter intervals, which has recently been shown to be inefficient if the goal were to maximise utilisation [75], showing again that cycle hire station supply does not necessarily lead to cycling demand. This also implies consistent supply over the operational area of the TfL cycle hire scheme in central London, further weakening the case for an implicit supply-demand consequence. From this, we conclude that there is no theoretical argument for a direct causal relationship between cycling infrastructure emergence and local business emergence, but rather that this effect—if there is any—is channelled via cycling activity. More generally, using infrastructure measure IVs is common practice in economics as they pose exogenous shocks to the system of interest (see e.g. [76,77]).

*Instrument III*: Bicycle accident data comes at LSOA level as counts of road accidents involving bicycles. The argument here is more abstract: new bicycle infrastructure and increased cycling usage initiate a “virtuous cycle” [78] of cycling availability, pro-cycling policies larger mode shares which in turn increase cycling safety and eventually reduce accidents involving cyclists. Here, the assumption of uncorrelated error terms is more intuitive. We also find no literature addressing causality between changes in cycling accidents and local businesses.

*Instrument IV*: The last instrument we suggest is count data for bicycle shops, which is also obtained with the amenity data obtained from OSM. Accordingly, we exclude bicycle shops from the overall shop count, our dependent variable. We argue that an increase in cycling infrastructure promotes the growth of private businesses related to cycling. To the best of our knowledge, there is no current literature confirming this hypothesis, however we believe that this idea is quite straightforward. Also, the exclusion restriction seems plausible. While bike shop growth might be correlated to general business growth in some places, it is truly driven by demand, i.e. cyclists as potential customers. Ideal locations for bike shops are hence easily accessible by bike, e.g. in more residential areas or close to popular cycling routes. In contrast, other shops like supermarkets or clothing stores will chose locations in malls or along busy roads where high footfall is expected, but which are not necessarily comfortably reached by bike.

To validate IV choice, we apply Pearson correlation tests, which can be shown to work for non-normally distributed observations, given a sufficient samples size (see Table 3).

**Table 3:** Pearson correlation tests between endogenous variable and potential IVs.

	Treatment		
Potent. IV	*Difference in cycle hire trip ends*		
	Cor. coeff.	t-statistic	p-value
Δ Cyc. hire stat. (TfL)	0.131	9.159	<0.001***
Δ Cyc. parking facil.	0.167	11.785	<0.001***
Δ Cyc. acc.	0.079	5.524	<0.001***
Δ Cyc. shops	0.128	8.942	<0.001***

Significance codes: 0 *** 0.001 ** 0.01 * 0.05.

*Note*: The Pearson tests are conducted with treatment dummies. The results with treatment intensities are not displayed as they don’t change the outcome significantly

We report correlation coefficient, t-statistic and the respective p-values for correlation tests between the treatment measure and each of the potential IV’s. We can see that all potential instruments are significantly correlated with the difference in cycle hire trip counts and thus pass the preliminary assessment.

### 4.4. Selection of exogenous control variables

We now seek to address possible bias introduced due to omitted variables. Previous literature addressing urban local business environments is widely available and justifies the use of measures that have proven to be related to an increase in consumption amenities. The most direct effect driving shop openings can be attributed to economic stimulus measures. For example, a recent study by Zheng *et al*. [79] name the emergence of local shops as a spillover effect of new industry park openings in China. Jardim [80] argues that the emergence of local retail and small businesses is a self-perpetuating process which can be exploited by policy interventions. Especially in cities, most public spending is concentrated on infrastructure with a large portion being allocated to the transportation sector. This requires the integration of some measure of wider public transport accessibility to control for the effect of large transport infrastructure projects on new shop openings. For London, this data is available in the form of the Public Transport Access Level (PTAL), as determined by TfL [81].

Beyond the public spending perspective, the characteristics of a neighbourhood reveal more connections with its respective local business environment, as new shop openings are intrinsically driven by projected profitability. Previous research has shown that vicinity income levels determine the distribution and emergence of consumption amenities: Wealthy neighbourhoods are more densely filled with supermarkets or convenience stores, while poor neighbourhoods exhibit more amenities related to alcohol consumption [82]. Furthermore, research has addressed the problem of ‘urban food deserts’, describing poor neighbourhoods with little access to quality food sources [83]. This suggests that education, labour or health statistics might be useful factors to investigate. Looking at socio-economic factors also seems relevant in the context of gentrification, i.e. the transformation of urban neighbourhoods due to changes in population characteristics and inflow of new, privileged citizens [84]. Gentrification sparks large scale restructuring of the built environment, along with rising housing prices which eventually drive away the previous, often structurally poorer and less educated residents. Griffith and Harmgart [85] note that densely populated areas produce more and smaller stores aimed at pedestrian retail shopping.

The available statistics provide sufficient characteristics to incorporate potential drivers of local business openings, thus preventing omission bias. We can access various social, economic and demographic measures as well as the above mentioned PTALs at LSOA level and select 12 exogenous variables, derived from literature, to be represented in the further modelling process. These measures are listed in Table 4, alongside their respective descriptive statistics.

**Table 4:** Descriptive statistics for selected exogenous predictor variables.

*Variable*	*mean*	*sd*	*median*	*se*
Pop. est. (2013)	1,740.75	304.55	1690	4.38
Pop. dens. (2013)	98.69	63.61	86	0.91
House price med. (2014)	444,375.09	32,3703	35,7800	4,655.31
PTAL avg. (2014)	3.74	1.6	3.3	0.02
Total No. children (2013)	364.84	149.27	350	2.15
Total No. road casualt. (2014)	6.36	8.98	4	0.13
% Pop. no qual. (2011)	17.84	7.33	17.6	0.11
% Pop. bad health (2011)	4.95	1.86	4.7	0.03
% HH no car (2011)	40.03	18.52	38.7	0.27
% Pop. unempl. (2011)	7.43	3.41	6.8	0.05
Med. income (2011)	35,756.46	11,459.9	32,609	164.81
Size (ha)	32.52	62.87	20.4	0.9

Note: Monetary measures are given as GBP (£); population is given as total numbers

n = 4835 observations

Note that since we rely on census data, the different statistics have been surveyed in varying years, ranging from 2011 to 2014. We include population counts, density measures and polygon size to reflect the basic structure of each LSOA. We add income, property prices and unemployment rate to represent the economic dimension. The number of children, education levels and health statistics reflect the socio-demographic dimension. Furthermore, we use public transport accessibility, car availability and road accident indicators for the local transportation environment. Lastly, public transport accessibility alongside LSOA size serves as approximation for the inner-city proximity of a neighbourhood, hence representing LSOA centrality in the model. Note here that, while we have also tested our models on inner-city LSOAs only (to where most of the cycle hire activity is confined), we have decided to include the full Greater London area, as our results were very similar and selection criteria for inner London are always to some degree arbitrary.

### 4.5. Model 1: 2-stage least squares (2SLS) regression

At this point, we have discussed all integral elements required for robust modelling, i.e. our outcome variable, the exogenous predictors and endogeneity treatment in the form of instrumental variables. The first modelling approach we test is a simple 2SLS regression. This method consists of two linear regression models and estimates a consistent IV estimator for the regression coefficient of our endogenous variable. Formally, we define the dependent variable *y*, a matrix of exogenous independent variables *x*_*EX*_, the endogenous independent variable *x*_*END*_ and lastly a matrix of instruments *z*. In the **first stage** of the 2SLS process, we estimate a linear model with the endogenous variable as a dependent variable and the IVs as independent variables

$${\hat{x}}_{i}^{END}=\delta_{0}+\delta_{1}z_{i}+\eta$$ Eq 5

where the estimated coefficient ${\hat{\delta }}_{1}=\frac{\sum_{i}z_{i}x_{i}}{\sum_{i}z_{i}^{2}}$ and *η* denotes the model error term.

The **second stage** uses the estimate ${\hat{x}}^{END}$ as independent variables in a linear model where our initial outcome *y* serves as independent variable:

$${\hat{y}}_{i}=\beta_{0}+\beta_{1}{\hat{x}}_{i}^{END}+\epsilon$$ Eq 6

The IV estimator is consistent and adjusted for endogeneity effects. Note that the 2SLS approach can be expanded to include further exogenous independent variables *x*^*EX*^ as control measures. The apparent problem with this modelling approach is the linear model assumption of a normally distributed error term *ϵ*. As discussed, we are operating in a non-normal environment. In fact, we have provided evidence that the outcome variable *y* follows a negative binomial distribution. The implications and limitations arising from this will be examined more thoroughly later in the paper. Beyond being non-normal, *ϵ* could also be non-independent, as our data exploration hints at the presence of spatial autocorrelation. However, since we have observed a strong correspondence of local spatial autocorrelation in the dependent variable with the centrality of London, we have some information on the underlying spatial process, helping us to mitigate some of the adverse effects. Evidently, the zero counts in our data also correspond to centrality, with almost no zero counts observed in central London. This implies that by accounting for the negative binomial nature of our count data, issues arising from spatial autocorrelation might be mitigated also. We explore this further in our discussion.

### 4.6. Model 2: 2-stage negative binomial (2SNB) regression

The second approach we test is an adaption of the 2-stage methodology for count data, where we deal with issues of non-normality and possible zero-inflation. We hereby follow the process outlined by [86]. Essentially, we repeat the **first stage** estimation introduced with the ordinary 2SLS. However, we replace the **second stage** linear model with a generalized linear model (GLM) that fits the observed negative binomial distribution.

If we recall section 4.1, we have defined the mean of a NB process as

$$\mu =\frac{rp}{1-p}$$ Eq 7

With $p=\frac{\mu }{\mu +x}$, so that we can formulate the probability mass function

$$f(x;r,p)\equiv \mathrm{Pr}\left(X=x\right)=\frac{\Gamma (r+x)}{k!\Gamma (r)}{\left(\frac{\mu }{r+\mu }\right)}^{x}{\left(\frac{r}{r+\mu }\right)}^{r}$$ Eq 8

Note that this formula is an analogous formulation to Eq 2, including the Γ parameter constituting the Poisson component of the NB distribution. We can expand Eq 8 to include a dispersion parameter $\alpha =\frac{1}{r}$ so that we can write the distribution as

$$\mathrm{Pr}\left(X=x\right)=\frac{\Gamma (\alpha^{-1}+x)}{k!\Gamma (\alpha^{-1})}{\left(\frac{\mu }{\alpha^{-1}+\mu }\right)}^{x}{\left(\frac{\alpha^{-1}}{\alpha^{-1}+\mu }\right)}^{\alpha^{-1}}$$ Eq 9

It can be shown that the NB distribution can be derived from a Poisson process, hence being also known as Poisson-gamma mixture. Accordingly, the traditional NB regression model can be written as

$$\ln\left(\mu \right)=\beta_{0}+\beta_{1}x$$ Eq 10

Where *μ* represents the mean of the outcome variable *y* while *x* represents the independent variables. As part of the independent variables, we include the fitted values from the *first stage* regression. The NB model parameters *β* and *α* can now be estimated via maximum likelihood (ML) estimation, where the likelihood function is given as:

$$L(\alpha ,\beta )=\prod_{i=1}^{n}p(y_{i})=\prod_{i=1}^{n}\frac{\Gamma (\alpha^{-1}+y_{i})}{\Gamma (\alpha^{-1})\Gamma (y_{i}+1)}{\left(\frac{\alpha e^{x_{i}\beta }}{\alpha e^{x_{i}\beta }+\mu }\right)}^{y_{i}}{\left(\frac{1}{1+\alpha e^{x_{i}\beta }}\right)}^{\alpha^{-1}}$$ Eq 11

The 2SNB approach is certainly more powerful when it comes to count data, however it also comes with restrictions and limitations in the applicable parametric tests. While we account for zero-inflated count data, any remaining RSA might still harm the explanatory power of the model. We thus explore this problem further, outlined in the discussion section. Our findings show that the effects of spatial autocorrelation are indeed substantially reduced by the NB approach, in some of the final models the effect becomes completely insignificant. Given these findings along with the methodological intricacies of the IV setting and the limited scope of this study, we conclude to not opt for an explicitly spatial model. In introducing the two regression models, we conclude the methodology section of this paper and proceed to reporting and discussing our empirical findings.

## 5. Results and discussion

### 5.1. Calibration of instrumental variables

We present our results in the same sequence as they were introduced previously, starting with Model 1, an ordinary 2SLS regression. We use the statistical programming language *R* (Version 3.4.1) for all data preparation and the statistical analysis. As outlined in section 4, we utilise a set of four potential instrumental variables, which all meet the basic requirement of a significant correlation with the endogenous predictor variable. However, this is not sufficient evidence of their fit as IVs. To identify the optimal IV configuration, we run three diagnostic tests within the 2SLS model. The first test is a simple F-test (also Wald test) to investigate instrument relevance. Our second test is the Wu-Hausman test, examining whether endogeneity is in fact an issue with our predictors (see [87]). The last test we run is the Sargan test assessing instrument validity for configurations applying more than one IV (see [88]). It can thus be used to analyse model overidentification. The results of our IV testing procedures are provided in Table 5.

**Table 5:** Instrument configuration testing for the endogenous variable Δ*Cyc*.*trip ends*.

	*Dependent variable*:					
	Δ Shops					
	(1) OLS	(2) 2SLS	(3) 2SLS	(4) 2SLS	(5) 2SLS	(6) 2SLS
*Endog*. *variable*	<0.000 *** (0.*00001)*	0.002 *** (0.*0005)*	0.011 *(0*.*006)*	0.004 *** (0.*001)*	<-0.000 *(0*.*0002)*	0.003 *** (0.*0004)*
Instruments	-	ΔCyc. hire stat.	ΔCyc. park. fac.	ΔCyc. shops	ΔCyc. acc.	ΔCyc. shops, (ΔCyc. hire stat+ΔCyc. park. fac.)
Wald test	-	13.93 ***	3.589	14.47 ***	21.297 ***	28.986 ***
Wu-Hausman test	-	31.69 ***	397.038 ***	195.81 ***	0.643	414.654 ***
Sargan test	-	-	-	-	-	2.759

Significance codes: 0 *** 0.001 ** 0.01 * 0.05.

Note: Selected exogenous variables (Table 4) are used in the models but not reported.

We see that for singular IV use, only difference in cycle hire stations and difference in bicycle shops pass both the Wald test and the Wu-Hausman test. After further calibration, we provide our optimal IV set in Model 6, a combination of the difference in bicycle shops and the sum of cycle hire station and bicycle parking facility differences. We denote this combined instrument as the difference in cycling infrastructure (Δ*Cyc*.*infr*. = Δ*Cyc*.*hire*.*stat*.+Δ*Cyc*.*park*.*fac*.). As we see, Model 6 passes all three tests including the Sargan test for multiple instruments. Note that the models are run including all exogenous predictors selected earlier, even though their estimates are not reported. We now apply the 2SLS method using the selected IV configuration to treat for endogeneity. Table 6 reports the results of the first stage.

**Table 6:** Regression results for the first stage of the 2SLS process using optimal IVs.

	*Dependent variable*:
	Δ Cyc. trip ends
*Independent variable*:	
Δ Cyc. shops	711.080** *(322*.*879)*
Δ Cyc. infr.	197.237*** *(29*.*947)*
Pop. est. (2013)	-0.714*** *(0*.*169)*
Pop. dens. (2013)	4.781*** *(0*.*842)*
House price med. (2014)	0.002*** *(0*.*0002)*
PTAL avg. (2014)	-295.099*** *(39*.*3)*
Total No. children (2013)	-0.29 *(0*.*52)*
Total No. road casualt. (2014)	138.444*** *(4*.*953)*
% Pop. no qual. (2011)	15.582 *(10*.*648)*
% Pop. bad health (2011)	-37.556 *(35*.*524)*
% HH no car (2011)	8.082* *(4*.*49)*
% Pop. unempl. (2011)	-38.832* *(22*.*088)*
Med. income (2011)	-0.005 *(0*.*007)*
Size (ha)	-2.861*** *(0*.*63)*
Constant	677.97 *(468*.*263)*
Observations	4,835
R^2^	0.247
Adjusted R^2^	0.245
Residual Std. Error	2,480.716 (df = 4820)
F Statistic	112.778*** (df = 14; 4820)

Significance codes: 0 *** 0.001 ** 0.01 * 0.05.

We see that both instruments significantly affect the endogenous variable Δ Cyc. trip ends. We also see that denser, smaller and economically prosperous areas exhibit more cycling trips. We now use the fitted values from the first stage for the estimation of the second stage model. In order to show the difference as compared to a model ignoring the endogeneity issue, we report the regression results of the 2SLS approach alongside a naïve OLS approach. Here, we also report a set of three dependent variables for the first time. As discussed in section 3.2, the OSM data we use to quantify local business amenities comes with various subcategories. Thus far, we have discussed all objects tagged as *shop*. However, to contextualise our research, we will also report results for a dependent variable denoting change in sustenance amenities (Δ Susten. amen.) and a combination of both categories (Δ Shops + Δ Susten. amen.).

### 5.2. Empirical findings

The results of the first approach (2SLS) are presented in Table 7.

**Table 7:** 2SLS regression results with optimal IVs.

	*Dependent variable*:					
	Δ Shops		Δ Susten. amen.		Δ Shops + Δ Susten. amen.	
*Independent variable*:	(1) OLS	(2) 2SLS	(3) OLS	(4) 2SLS	(5) OLS	(6) 2SLS
Δ Cyc. trip ends	0.0001***	0.003***	0.0001***	0.0004***	0.0002***	0.003***
	*(0*.*00002)*	*(0*.*0004)*	*(0*.*00000)*	*(0*.*00005)*	*(0*.*00002)*	*(0*.*0004)*
Pop. est. (2013)	0.001***	0.003***	-0.0001***	0.0001	0.001***	0.003***
	*(0*.*0002)*	*(0*.*001)*	*(0*.*00002)*	*(0*.*0001)*	*(0*.*0002)*	*(0*.*001)*
Pop. dens. (2013)	-0.004***	-0.016***	0.0004***	-0.001***	-0.004***	-0.017***
	*(0*.*001)*	*(0*.*003)*	*(0*.*0001)*	*(0*.*0004)*	*(0*.*001)*	*(0*.*003)*
House price med. (2014)	0.00000**	-0.0000***	-0.00000***	-0.0000***	0.00000	-0.0000***
	*(0*.*00000)*	*(0*.*00000)*	*(0*.*00000)*	*(0*.*00000)*	*(0*.*00000)*	*(0*.*00000)*
PTAL avg. (2014)	0.255***	1.014***	-0.015***	0.075***	0.240***	1.089***
	*(0*.*042)*	*(0*.*157)*	*(0*.*006)*	*(0*.*019)*	*(0*.*043)*	*(0*.*174)*
Total No. children (2013)	-0.002***	-0.001	0.0001*	0.0002	-0.002***	-0.001
	*(0*.*001)*	*(0*.*002)*	*(0*.*0001)*	*(0*.*0002)*	*(0*.*001)*	*(0*.*002)*
Total No. road casualt. (2014)	0.068***	-0.335***	0.022***	-0.026***	0.090***	-0.361***
	*(0*.*005)*	*(0*.*059)*	*(0*.*001)*	*(0*.*007)*	*(0*.*006)*	*(0*.*065)*
% Pop. no qual. (2011)	0.028**	-0.014	0.0001	-0.005	0.028**	-0.019
	*(0*.*011)*	*(0*.*032)*	*(0*.*002)*	*(0*.*004)*	*(0*.*011)*	*(0*.*035)*
% Pop. bad health (2011)	-0.136***	-0.041	-0.006	0.006	-0.142***	-0.036
	*(0*.*038)*	*(0*.*104)*	*(0*.*005)*	*(0*.*013)*	*(0*.*038)*	*(0*.*115)*
% HH no car (2011)	0.029***	-0.001	0.001	-0.003*	0.029***	-0.004
	*(0*.*005)*	*(0*.*014)*	*(0*.*001)*	*(0*.*002)*	*(0*.*005)*	*(0*.*015)*
% Pop. unempl. (2011)	-0.045*	0.075	-0.004	0.010	-0.049**	0.085
	*(0*.*023)*	*(0*.*066)*	*(0*.*003)*	*(0*.*008)*	*(0*.*024)*	*(0*.*074)*
Med. income (2011)	0.00000	0.00001	0.00000***	0.00001**	0.00001	0.00002
	*(0*.*00001)*	*(0*.*00002)*	*(0*.*00000)*	*(0*.*00000)*	*(0*.*00001)*	*(0*.*00002)*
Size (ha)	0.001	0.009***	-0.0003***	0.001**	0.0004	0.009***
	*(0*.*001)*	*(0*.*002)*	*(0*.*0001)*	*(0*.*0003)*	*(0*.*001)*	*(0*.*002)*
Constant	-2.269***	-3.435**	0.090	-0.048	-2.179***	-3.483**
	*(0*.*493)*	*(1*.*369)*	*(0*.*067)*	*(0*.*166)*	*(0*.*503)*	*(1*.*515)*
Adjusted R^2^	0.209	-	0.425	-	0.259	-
Residual Std. Error (df = 4821)	2.621	7.218	0.355	0.875	2.673	7.986
F Statistic (df = 13; 4821)	99.108***	-	275.448***	-	130.778***	-
Wald test	-	28.986***	-	28.986***	-	28.986***
Wu-Hausman test	-	414.654***	-	313.334***	-	507.94***
Sargan test	-	2.759	-	0.068	-	2.17

Significance codes: 0 *** 0.001 ** 0.01 * 0.05.

The first thing we note is that the endogenous variable is consistently positive and significant, for both ordinary OLS and the 2SLS approach. The 2SLS estimates for Δ Cyc. trip. ends are 0.003 (Dep. var. = Δ Shops) and 0.0004 (Dep. var. = Δ Susten. amen.) and suggest that it takes about 333 more cycling trips within a LSOA for a new shop to emerge and about 2500 more cycling trips for a new sustenance amenity to emerge (within our observed timeframe). For the Models 1 and 2, we see that the significant effects of total population and population density barely change between OLS and 2SLS. When switching from OLS to 2SLS, the effect of public transport accessibility is heavily boosted, while the estimate of total road casualty changes from positive to negative. Population health, number of children and number of households without a car lose their significance when moving to 2SLS while LSOA size surpasses the significance threshold. Moving to the next dependent variable, Models 3 and 4 behave similarly with the difference of median income being highly (positively) significant for both, the OLS and 2SLS model. The combined Models 5 and 6 are again very close to Models 1 and 2. When looking at the 2SLS models only, we see that across the board cycling trip ends and public transport accessibility have a positive effect on the respective dependent variable. This confirms our hypothesis that the transportation ecosystem—cycling specifically and also in the broader sense—positively affects the economic environment and hence promotes new local business openings. Furthermore, we see that population density, median house price and total road casualties negatively affect all dependent variables. All models come with diagnostic statistics in the form of the coefficient of determination *R*^2^ (we only report the adjusted *R*^2^ value which accounts for degrees of freedom in the model) and the residual standard error. 2SLS regressions allow *R*^2^ computations, however they have no statistical meaning and are hence not reported. Although the 2SLS approach delivers interesting results, the explanatory power of the first model is limited. Since this ordinary IV method is applied using OLS estimators, we violate the critical assumption of normally distributed error terms as our data stems from a negative binomial process. The model residuals behave accordingly, which is confirmed by Shapiro-Wilk normality tests and furthermore discussed in section 4.

We now turn to the alternative approach, which replaces the second stage of the 2SLS with a negative binomial regression. We refer to this adapted approach as 2SNB. Again, our results are reported for the three different response variables, as displayed in Table 8.

**Table 8:** 2SNB regression results with optimal IVs.

	*Dependent variable*:		
	Δ Shops	Δ Susten. amen.	Δ Shops+ Δ Susten. amen.
*Independent variable*:	(1) 2SNB	(2) 2SNB	(3) 2SNB
Δ Cyc. trip ends	0.001***	0.0003***	0.001***
	*(0*.*0001)*	*(0*.*0001)*	*(0*.*0001)*
Pop. est. (2013)	0.002***	0.001*	0.002***
	*(0*.*0002)*	*(0*.*0003)*	*(0*.*0002)*
Pop. dens. (2013)	-0.009***	-0.013***	-0.009***
	*(0*.*001)*	*(0*.*002)*	*(0*.*001)*
House price med. (2014)	-0.00000***	-0.00000***	-0.00000***
	*(0*.*00000)*	*(0*.*00000)*	*(0*.*00000)*
PTAL avg. (2014)	0.686***	0.400***	0.685***
	*(0*.*056)*	*(0*.*098)*	*(0*.*055)*
Total No. children (2013)	0.0002	0.001	0.0002
	*(0*.*001)*	*(0*.*001)*	*(0*.*001)*
Total No. road casualt. (2014)	-0.170***	-0.037**	-0.170***
	*(0*.*020)*	*(0*.*016)*	*(0*.*019)*
% Pop. no qual. (2011)	-0.033**	-0.049	-0.033***
	*(0*.*013)*	*(0*.*039)*	*(0*.*013)*
% Pop. bad health (2011)	-0.001	0.040	-0.003
	*(0*.*041)*	*(0*.*097)*	*(0*.*040)*
% HH no car (2011)	0.016***	0.075***	0.017***
	*(0*.*005)*	*(0*.*012)*	*(0*.*005)*
% Pop. unempl. (2011)	0.006	-0.050	0.005
	*(0*.*026)*	*(0*.*066)*	*(0*.*026)*
Med. income (2011)	0.00001	0.00005***	0.00001*
	*(0*.*00001)*	*(0*.*00002)*	*(0*.*00001)*
Size (ha)	0.005***	0.001	0.005***
	*(0*.*001)*	*(0*.*004)*	*(0*.*001)*
Constant	-4.723***	-9.841***	-4.731***
	*(0*.*533)*	*(1*.*185)*	*(0*.*522)*
Log Likelihood	-4,204.856	-435.506	-4,278.978
*θ*	0.186*** (0.009)	1.086** (0.466)	0.195*** (0.009)
AIC	8,437.712	899.011	8,585.956

Significance codes: 0 *** 0.001 ** 0.01 * 0.05

Once more, cycling trips are highly significant while the estimates show a strong resemblance with the results obtained from the 2SLS models, although the output of the NB regressions is interpreted differently. The estimated coefficients describe the change in the difference in the logs of mean counts for the dependent variable, given a one-unit change in the respective independent variable. We estimate 2SNB coefficients of 0.001 (for dep. var. = Δ Shops) and 0.0003 (for dep. var. = Δ Susten. amen.) for the change in cycle trip ends. As for the other exogenous predictors, the NB approach appears rather consistent across the three dependent variables. Total population, public transport accessibility and the number of households with no cars all have a positive effect on the dependent variable for the Models 1, 2 and 3. Population density, median house price and total road casualties exhibit negative effects across the board. Differences arise in population percentage without qualification, which has a negative effect in Models 1 and 3, but not in Model 2. Similarly, LSOA size has a positive effect in Models 1 and 3, but not in 2. Lastly, median income has a significant positive impact in Models 2 and 3, however, it is insignificant in Model 1. These results correspond with the 2SLS approach, yet less precisely with naïve OLS. The strongest discrepancy between 2SLS and 2SNB is observed in the predictor denoting the percentage of households without a car. While this estimate is inconsistently significant and mostly negative in the 2SLS models, it exhibits a consistently positive and significant effect in all 2SNB models. Overall, our models seem to be able to estimate sustenance amenity emergence substantially better than general shops, as *R*^2^ and Akaike Information Criterion (AIC) values indicate.

We report three diagnostics for each of the 2SNB models: Log likelihood, AIC and *θ* value. The log likelihood refers to our model estimation via maximum likelihood. The AIC, first introduced by Akaike [89], abstractly describes the information loss of a given model when compared to the original process. As a rule of thumb, the best model is always the model with an AIC closer to zero, as this indicates less information loss. The last testing procedure we undergo to confirm goodness-of-fit is a test for overdispersion. This helps us to assess, whether the NB model is in fact the right choice, as opposed to a regular Poisson model. The idea for the test was introduced by Dean [90] and has since been applied in different forms and is discussed enthusiastically within the scientific community (e.g. [91]). We test for the null hypothesis *H*_0_:*θ* = 0, i.e. that we are actually dealing with a Poisson model, and display our results in Table 9. In all three models, we clearly reject the null hypothesis of a true Poisson model, thus validating the NB model choice. The exact *θ* estimates with standard errors are reported with the 2SNB regression results in Table 8.

**Table 9:** Likelihood ratio test results for 2SNB models.

	*Dependent variable*:		
	Δ Shops	Δ Susten. amen.	Δ Shops + Δ Susten. amen.
	(1) 2SNB	(2) 2SNB	(3) 2SNB
*Likelihood ratio test*	6509.1412***	6.9623***	6472.0004***

Significance codes: 0 *** 0.001 ** 0.01 * 0.05

**Fig 6:**
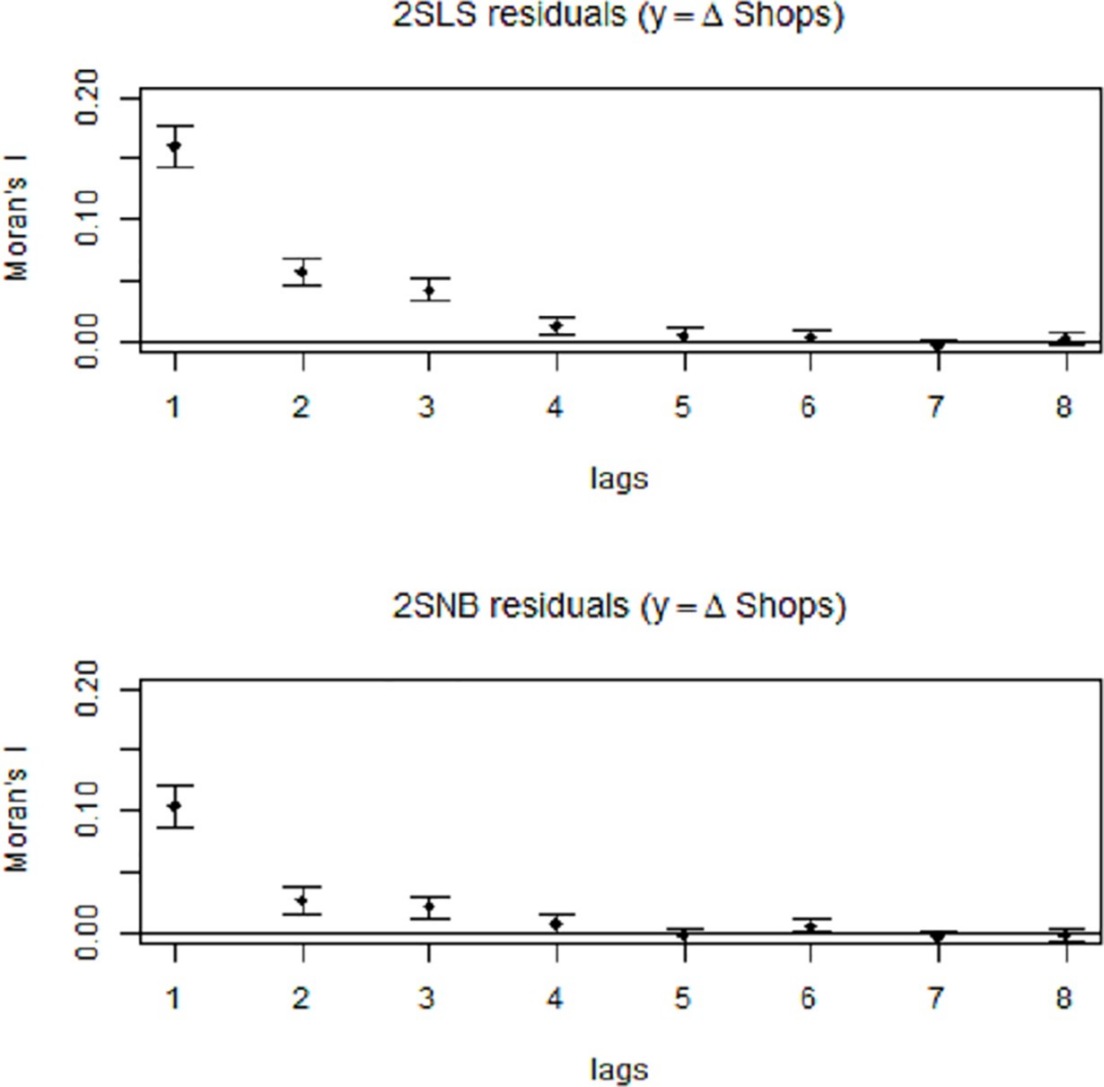
Spatial correlograms using Local Moran’s I values of model residuals for the 2SLS and the 2SNB model, with dependent variable Δ*Shops*.

Lastly, we investigate potential RSA in our models. We apply global and local Moran’s I tests to the residuals from our 2SLS and 2SNB models. Our findings show that global RSA is present in all 2SLS models. For the 2SNB models, only the y = Δ Shops and y = (Δ Shops + Δ Susten. amen.) models exhibit significant global RSA. We further assess local RSA for both of these models by computing correlograms. This allows us to examine how the distance between a pair of observations affects their correlation. As expected—and shown in Fig 6—we find that the 2SNB model substantially mitigates RSA, making the effect negligible after a lag of one spatial unit (one LSOA). From this we infer that the spatial dependencies observed are to a large degree due to the centrality bias in our data, which we account for using a negative binomial model. This is confirmed by looking into the spatial distribution of the local Moran’s I values for the model residuals: While the 2SLS residuals are mostly significantly autocorrelated in Central London, the 2SNB residuals only exhibit autocorrelation in a few hot-spots. While these hot-spots warrant a closer examination into the spatial dynamics in future research, the effect seems to be rather small—especially in the 2SNB setting. Overall, we believe that it does not substantially affect the consistency and robustness of our estimator. While limitations apply, our findings provide a first evidence for a causal link between increased cycling activity and local business openings.

## 5.3. Discussion

Before we sum up our study, we address the contributions and limitations of our work. This study offers an entirely new perspective on interactions between cycling and local businesses, thus improving the current state of research in the field. Our findings might not only motivate future studies but also help the public and private sector gain a deeper understanding of the economic effects of cycling, thus enhancing planning and appraisal procedures. Nevertheless, we discuss some limitations of our methodology. First, our data processing relies on some underlying assumptions about OSM data quality, mainly that OSM tagging dates approximate the true opening dates of shops and consumption amenities. This and other data biases, like an overrepresentation of wealthy, well-educated subpopulations, is discussed in previous sections of the paper. Overall, however, we do believe that there is currently no better data provider for our particular question. As we have highlighted, the 2SLS approach is flawed for assuming normally distributed data in a negative binomial environment. Still, previous research has shown that applying OLS estimation does not yield more false-positive values than expected [92] and hence still provides substantial insights. Despite this, the 2SNB model overall still provides the better fit. In terms of the data basis, our model might suffer from distortion, as it has been shown that excessive outlier occurrence renders maximum likelihood estimation inconsistent, requiring more robust estimation techniques [93]. Even though we account for outliers, as a further methodological enhancement, zero-inflated models could be tested. Alternatively, sample exclusion or difference-in-difference approaches might also tackle this concern. Another potential issue throughout the modelling process are spatial interdependencies between neighbouring LSOAs. We do find some degree of spatial autocorrelation to be present in our model. Even though the 2SNB model appears to capture most of this effect, this does influence the certainty with which we can draw conclusions about causality. While we incorporate spatial densities, we exclude the spatial spillover effects in our methodological framework. Notably, temporal autocorrelation is not an issue since we explicitly work with difference over time, rather than yearly point-in-time extracts. Overall, this study is only the first step into a new research direction and cannot address all potential challenges within its limited scope. We hence want to encourage researchers to build on our findings, to improve methodology and to address related questions.

## 6. Conclusion

In this paper, we have sought to isolate the effect of increased cycling activity on the openings of new local businesses, specifically shops and sustenance amenities. We have compiled a novel dataset from London, a city where cycling still plays a tangential role yet experiences increasing adoption—particularly since the introduction of Transport for London’s cycle hire scheme. We have used this bike-sharing data to quantify cycling activity, along with further measures of the cycling ecosystem: the location counts of cycle hire stations, bicycle parking facilities, bicycle shops and bicycle accidents. Furthermore, we have gathered locations for local business amenities. We have aggregated point data, cycling activity data and accident data at the level of 4835 Lower Super Output Areas in Greater London. This has enabled us to merge the data with several economic and socio-demographic statistics. All our sources are publicly available open data archives. The London Datastore has provided geographical shapes and census statistics for each LSOA, TfL has provided extensive cycling trip and accident data and the community mapping service OpenStreetMap has provided point location data for physical amenities such as cycle hire stations, bicycle parking facilities, shops or restaurants. First, we have shown that our data fits a negative binomial distribution, a typical behaviour for count data. We have then developed a methodological framework to estimate the causal effect of cycling on the emergence of new local businesses. We have proven that LSOAs which experience an increase in cycling activity also exhibit higher numbers of new local business openings, using graphical comparison and a bootstrapped distribution test. Constructing the modelling approach, we have identified and treated two endogeneity issues: first by introducing instrumental variables to account for the reverse causality between increased cycling and increased business amenity counts. Secondly, we have avoided omitted variable bias by incorporating additional factors that might drive local business growth in the model.

We have introduced two models for parameter estimation: (1) an ordinary two-stage least squares model and (2) a two-stage negative binomial model. We have shown that the 2SLS approach lacks fit, as it disregards the non-normal nature of our data. Calibration of the IV configuration has indicated a combination of change in bicycle shops and the sum of change in cycle hire stations and change in bicycle parking facilities as the optimal IV set. Those instruments have then been used in the regression frameworks with change in local business amenities as a dependent variable, change in bicycle trips as an endogenous predictor and 12 selected socio-economic factors as exogenous predictors. For all estimated IV models, the change of cycling trips has been highly significant and has positively affected change in local businesses. Furthermore, we have observed that areas with good public transport accessibility, less private car ownership and high total population have significantly stronger local business amenity growth, while low education level and high population density have negatively affected the emergence of new local businesses. Finally, we have validated NB model choice by testing for overdispersion in the model. The key finding of our study seem relatively robust, as the estimated parameters only vary marginally across the models.

Our analysis has entailed some assumptions and restrictions. First and foremost, our data basis might have been biased. Since OSM is based on volunteered information, it is not always precise and timely. In our model, we have assumed that the point-in-time an object was tagged on OSM approximates the actual time of emergence (i.e. opening time, construction time). Nonetheless, previous research has shown OSM data to be of relatively high quality. As a second restriction, choosing instruments is always challenging. Here, we have assumed that our measures of the cycling ecosystem correlate with cycling activity sufficiently well while not being caused by the same factors. The TfL cycling strategy provides evidence for this claim. Lastly, our modelling approaches—while reflecting the distributional nature of our data—have been impaired by potential inconsistency in maximum likelihood estimation and spatial autocorrelation. While we find these effects to be marginal, we still want to highlight that they have to be considered when evaluating the findings of our study. Finally, we suggest some fruitful directions for future work in this area. For the most part, research on cycling and the urban environment crucially depends on the quality of data. Even though OSM is certainly the best available option, more precise datasets could validate previous findings or generate new insights. Unfortunately, many useful data sources are still proprietary. We can only speculate how much our analysis would have profited from reliable mapping data as gathered by companies like Google or HERE. Regarding our methodology, future work may incorporate spatial interaction models like geographically weighted regression (GWR) or spatial lag models. Especially in the urban setting with large and often highly granular datasets, statistical learning methods might also prove helpful.
